# Modeling and estimation of physiochemical properties of cancer drugs using entropy measures

**DOI:** 10.1038/s41598-025-87755-5

**Published:** 2025-02-08

**Authors:** Qasem M. Tawhari, Muhammad Naeem, Abdul Rauf, Muhammad Kamran Siddiqui, Oladele Oyelakin

**Affiliations:** 1 Department of Mathematics, College of Science, Jazan University, Jazan, 45142 Saudi Arabia; 2 Department of Mathematics, National University of Sciences and Technology (NUST), Islamabad, Pakistan; 3 Department of Mathematics, Air University Multan Campus, Multan, Pakistan; 4 Department of Mathematics, Comsats University Islamabad, Lahore, Pakistan; 5 Chemistry Unit, School of Arts & Sciences, University of The Gambia, Serrekunda, The Gambia

**Keywords:** Physiochemical characteristics, QSPR Analysis, drugs, Topological indices, Reverse entropy measures, Computational science, Cheminformatics

## Abstract

Hyaluronic acid-paclitaxel conjugate is a nanoparticle-based drug delivery system that combines hyaluronic acid with paclitaxel, enhancing its solubility, stability, and targeting specificity. This conjugate shows promise in treating breast, lung, and ovarian cancers with reduced side effects. Entropy measures are used to predict physical and chemical properties of drugs. In this paper, we compute entropy measures for the hyaluronic acid-paclitaxel conjugate using the edge/connectivity partition approach. We establish a quantitative structure-property relationship using reverse entropy measures to predict physical properties of cancer drugs. Multiple linear, Ridge, Lasso, ElasticNet, and Support Vector regression models are employed using Python software. Our results show that reverse entropy measures exhibit high predictive capability for physical properties, based on the highest coefficient of determination and lowest mean squared error. We conclude that physical properties, including boiling point, enthalpy of vaporization, flash point, molar refractivity, molar volume, polarization, molecular weight, monoisotopic mass, topological polar surface area, and complexity, can be predicted using reverse entropy measures. We propose models for each relationship, including only the most significant models for estimating uncalculated physical properties.

## Introduction

   The mathematical field of chemical graph theory focuses on the study of chemical graphs, which are mathematical structures representing pairwise interactions between entities^1^. A graph consists of two primary components: edges (bonds) and vertices (atoms), also referred to as nodes. In this representation, vertices denote atoms, while edges (bonds) represent the interactions between them^2^.

Mathematical chemistry, a subfield of theoretical chemistry, employs mathematical methodologies to investigate and understand the properties and dynamics of chemical compounds^3,4^. Topological indices are numerical values corresponding to the molecular structure of compounds. Various topological indices have been defined, including degree-based, eigenvalue-based, and distance-based indices^5^. Researchers have found applications for these indices in chemistry, pharmacy, and biology^6^.

The concept of topological indices was first introduced by Wiener^7^. Later, Milan Randić^8^ proposed the Randić index. Amic et al.^9^ and Bollobas et al.^10^ subsequently suggested a generalization of the Randić index. In 2010, Trinajstić and Zhou introduced the sum connectivity index^11^. Zhong^12^ defined the harmonic index in 2012. Nikolic et al.^13^ presented a revised version of the second Zagreb index, while Fath-Tabar^14^ introduced the third Zagreb index in 2011. In 2015, Munir et al.^15^ converted degree-based indices into entropy measures. Ranjini et al.^16^ proposed revised Zagreb indices in 2013. Notably, Shannon^17^ laid the foundation for entropy metrics in 1948.

Researchers initiated investigations into the entropy value of network systems in the late 1950s, inspired by Shannon’s influential work^18^. Rashevsky employs the concept of entropy measures to quantify the structural complexity of a graph. Shannon’s entropy is employed to quantify the intricacy of the graph in this particular scenario. Mowshowitz^19^ subsequently conducted an examination of the characteristics of graph entropy and conducted extensive measurements pertaining to his particular application. Entropy indicators based on graphs have been employed in diverse disciplines such as biology, chemistry, and computer science for the purpose of characterizing patterns^20^. The entropy proposed by Korners^21^ serves as another notable illustration.

Entropy measures have been developed using several graph invariances, including eigenvalues and connection information^22^, degree-based graph entropy^23^, and distance-based graph entropy^24^. Kulli introduced the concept of reverse degree-based indices^25^. Many researchers calculate the reverse degree-based indices for certain chemical structures. Wei Gao et al. calculated the indices for dendrimers^26^. Koam et al. calculated the indices for third type of chain hex-derived network^27^. Dongming Zhao et al. examine the polycyclic metal-organic network^28^. For more information about reverse degree-based indices calculations for certain chemical structures, sees^29–31^.

Recently, Furtula et al.^32^ did an analysis of the popular degree-based indices using the data of the octane isomer. They concluded that the symetric division degree index is the descriptor. Recently, Rauf et al.^33^ developed the QSPR between degree-based entropy and physical properties. They show that entropy measures are helpful in predicting physical properties. QSPR is a highly effective analytical methodology utilized to transform a given molecule into a series of numerical values that accurately depict its intrinsic chemical and physical characteristics. Several researchers have conducted studies on statistical linear, quadratic, and polynomial regression models to analyze the relationship between indices and physical properties^34–36^.

Motivated by these authors’ works, we have developed the QSPR between the physical properties of cancer drugs and reverse degree-based entropy measures. We found that the entropy measures show the best correlation with physical properties. These results motivate and are helpful for researchers in predicting physical properties.

### Degree based entropy of a graph

Let $Z=(V,E)$ be a finite, simple, and connected graph of order *p*, size *q* edges and $\mathscr {F}$ be a real valued function. The **degree** of a vertex *r* is denoted by *d*(*r*) and is defined as the number of edges attached with vertex *r*. Kulli^37^ introduced the **reverse degree** $\Upsilon (r)$ defined as $\Upsilon (r)= \bigtriangleup (Z)-d(r)+1$, where $\bigtriangleup (Z)$ is the maximum degree of vertex among the vertices of a graph *Z*. For the undefined term of graph theory, see^38^. The entropy function of graph *Z* is defined as follows:

1 $${\mathcal{E}}_{{\mathcal{F}}} (Z) = - \sum\limits_{{i = 1}}^{p} {\frac{{{\mathcal{F}}(r_{i} )}}{{\sum\nolimits_{{j = 1}}^{p} {\mathcal{F}} (r_{j} )}}} log\left[ {\frac{{{\mathcal{F}}(r_{i} )}}{{\sum\nolimits_{{j = 1}}^{p} {\mathcal{F}} (r_{j} )}}} \right].$$

Now, if $r_i\in V$ and $\mathscr {F} (r_i)$ is an information function that represent the degree of vertex $r_i$, denoted by $\mathscr {F} (r_i)=\Upsilon (r_i)$, then equation (1) becomes

$${\mathcal{E}}_{{\mathcal{F}}} (Z) = - \sum\limits_{{i = 1}}^{p} {\frac{{\Upsilon (r_{i} )}}{{\sum\nolimits_{{j = 1}}^{p} \Upsilon (r_{j} )}}} log\left[ {\frac{{\Upsilon (r_{i} )}}{{\sum\nolimits_{{j = 1}}^{p} \Upsilon (r_{j} )}}} \right],$$

$$\mathscr {E}_{\mathscr {F}} (Z)=log \left(\sum ^p_{j=1}\Upsilon (r_j)\right)-\frac{1}{(\sum ^p_{j=1}\Upsilon (r_j))}\sum ^p_{i=1}[\hbar (r_i)log(\Upsilon (r_i))].$$

By simplifying the equation and using $\sum ^n_{j=1}\Upsilon (r_j)=2q$,

2 $${\mathcal{E}}_{{\mathcal{F}}} (Z) = log(2q) - \frac{1}{{2q}}log\left[ {\prod\limits_{{i = 1}}^{p} \Upsilon (r_{i} )^{{\Upsilon (r_{i} )}} } \right].$$

### Edge weight-based entropy of graph

In 2014, Chen et al. introduced the concept of the entropy of an edge weight graph. For an edge weight graph $P=(V(Z);E(Z):\mathscr {F}(rs))$, where *E*(*Z*) is the edge set, *V*(*Z*) is the vertex set, and $\mathscr {F}(rs)$ is the edge weight of the edge *rs* in *Z*. The entropy is defined as

3 $${\mathcal{E}}_{{\mathcal{F}}} (Z) = - \sum\limits_{{r^{\prime } s^{\prime } \in E(Z)}} {\frac{{{\mathcal{F}}(r^{\prime } s^{\prime } )}}{{\sum\nolimits_{{rs \in E(Z)}} {{\mathcal{F}}(rs)} }}} log\left[ {\frac{{{\mathcal{F}}(r^{\prime } s^{\prime } )}}{{\sum\nolimits_{{rs \in E(Z)}} {{\mathcal{F}}(rs)} }}} \right].$$

By using Table 1 and simplifying the equation (3), we can derive the entropy measures written in Table 2.

**Table 1:** Notation and formulas of the reverse degree based indices.

Name of Index	Notation	Formula	Formula expansion
Randić $\alpha =-1, \frac{-1}{2}, 1, \frac{1}{2}$	$R_{\alpha }(Z)$	$\sum _{rs\in E (Z)} \mathscr {F}_{ R_{\alpha }}(rs)$	$\sum _{rs\in E (Z)} ({\Upsilon }_r\times {\Upsilon }_s)^\alpha$
Atom bond connectivity	*ABC*(*Z*)	$\sum _{rs\in E (Z)} \mathscr {F}_{ABC}(rs)$	$\sum _{rs\in E (Z)}\sqrt{\frac{{\Upsilon }_r+{\Upsilon }_s-2}{{\Upsilon }_r\times {\Upsilon }_s}}$
Geometric arithmetic	*GA*(*Z*)	$\sum _{rs\in E (Z)} \mathscr {F}_{GA}(rs)$	$\sum _{rs\in E (Z)} \frac{2\sqrt{{{\Upsilon }_r\times {\Upsilon }_s}}}{{{\Upsilon }_r+ {\Upsilon }_s}}$
First Zagreb	$M_1 (Z)$	$\sum _{rs\in E (Z)} \mathscr {F}_{M_1}(rs)$	$\sum _{rs\in E (Z)}({\Upsilon }_r+{\Upsilon }_s)$
Second Zagreb	$M_2 (Z)$	$\sum _{rs\in E (Z)} \mathscr {F}_{M_2}(rs)$	$\sum _{rs\in E (Z)}({\Upsilon }_r\times {\Upsilon }_s)$
Hyper Zagreb	*HM*(*Z*)	$\sum _{rs\in E (Z)} \mathscr {F}_{HM}(rs)$	$\sum _{rs\in E (Z)}({\Upsilon }_r+{\Upsilon }_s)^2$
Forgotten	*F*(*Z*)	$\sum _{rs\in E (Z)} \mathscr {F}_{F}(rs)$	$\sum _{rs\in E (Z)}[({\Upsilon }_r)^2+({\Upsilon }_s)^2]$
Augmented Zagreb	*AZI*(*Z*)	$\sum _{rs\in E (Z)} \mathscr {F}_{AZI}(rs)$	$\sum _{rs\in E (Z)} (\frac{{\Upsilon }_r\times {\Upsilon }_s}{{\Upsilon }_r+ {\Upsilon }_s-2})^3$
Balaban	*J*(*Z*)	$\sum _{rs\in E (Z)} \mathscr {F}_{J}(rs)$	$\sum _{rs\in E (Z)} (\frac{q}{q-p+2}\times \frac{1}{\sqrt{{\Upsilon }_r\times {\Upsilon }_s}})$
Redefined first Zagreb	$ReZG_1 (Z)$	$\sum _{rs\in E (Z)} \mathscr {F}_{ReZG_1}(rs)$	$\sum _{rs\in E (Z)}\frac{{\Upsilon }_r+{\Upsilon }_s}{{\Upsilon }_r\times {\Upsilon }_s}$
Redefined second Zagreb	$ReZG_2 (Z)$	$\sum _{rs\in E (Z)} \mathscr {F}_{ReZG_2}(rs)$	$\sum _{rs\in E (Z)}\frac{{\Upsilon }_r\times {\Upsilon }_s}{{\Upsilon }_r+{\Upsilon }_s}$
Redefined third Zagreb	$ReZG_3 (Z)$	$\sum _{rs\in E (Z)} \mathscr {F}_{ReZG_3}(rs)$	$\sum _{rs\in E (Z)}({\Upsilon }_r\times {\Upsilon }_s)({\Upsilon }_r+ {\Upsilon }_s)$

**Table 2:** Notation and formulas of entropy measures.

Name of entropies	Notation	Formula
Randić $\alpha =-1, \frac{-1}{2}, 1, \frac{1}{2}$	$\mathscr {E}_{\alpha }(Z)$	$log(R_{\alpha })-\frac{1}{R_{\alpha }}log \left[ \prod _{rs\in E(Z)} [\mathscr {F}_{ R_{\alpha }}(rs)]^{ \mathscr {F}_{ R_{\alpha }}(rs) }] \right]$
Atom bond connectivity	$\mathscr {E}_{ABC}(Z)$	$log(ABC)-\frac{1}{ABC}log\left[ \prod _{rs\in E(Z)} [\mathscr {F}_{ABC}(rs)]^{ \mathscr {F}_{ABC}(rs)}\right]$
Geometric arithmetic	$\mathscr {E}_{GA}(Z)$	$log(GA)-\frac{1}{GA}log\left[ \prod _{rs\in E(Z)} [\mathscr {F}_{GA}(rs)]^{ \mathscr {F}_{GA}(rs)}\right]$
First Zagreb	$\mathscr {E}_{M_1}(Z)$	$log(M_1)-\frac{1}{M_1}log\left[ \prod _{rs\in E(Z)} [\mathscr {F}_{M_1}(rs)]^{ \mathscr {F}_{M_1}(rs)}\right]$
Second Zagreb	$\mathscr {E}_{M_2}(Z)$	$log(M_2)-\frac{1}{M_2}log\left[ \prod _{rs\in E(Z)} [\mathscr {F}_{M_2}(rs)]^{( \mathscr {F}_{M_2}(rs)}\right]$
Hyper Zagreb	$\mathscr {E}_{HM}(Z)$	$log(HM)-\frac{1}{HM}log\left[ \prod _{rs\in E(Z)} [\mathscr {F}_{HM}(rs)]^{ \mathscr {F}_{HM}(rs)}\right]$
Forgotten	$\mathscr {E}_{F}(Z)$	$log(F)-\frac{1}{F}log\left[ \prod _{rs\in E(Z)} [\mathscr {F}_{F}(rs)]^{ \mathscr {F}_{F}(rs)}\right]$
Augmented Zagreb	$\mathscr {E}_{AZI}(Z)$	$log(AZI)-\frac{1}{AZI}log\left[ \prod _{rs\in E(Z)} [\mathscr {F}_{AZI}(rs)]^{ \mathscr {F}_{AZI}(rs)}\right]$
Balaban	$\mathscr {E}_{J}(Z)$	$log(J)-\frac{1}{J}log\left[ \prod _{rs\in E(Z)} [\mathscr {F}_{J}(rs)]^{ \mathscr {F}_{J}(rs)}\right]$
Redefined first Zagreb	$\mathscr {E}_{ReZG_1}(Z)$	$log(ReZG_1)-\frac{1}{ReZG_1}log\left[ \prod _{rs\in E(Z)} [\mathscr {F}_{ReZG_1}(rs)]^{ \mathscr {F}_{ReZG_1}(rs)}\right]$
Redefined second Zagreb	$\mathscr {E}_{ReZG_2}(Z)$	$log(ReZG_2)-\frac{1}{ReZG_2}log\left[ \prod _{rs\in E(Z)} [\mathscr {F}_{ReZG_2}(rs)]^{ \mathscr {F}_{ReZG_2}(rs)}\right]$
Redefined third Zagreb	$\mathscr {E}_{ReZG_3}(Z)$	$log(ReZG_3)-\frac{1}{ReZG_3}log\left[ \prod _{rs\in E(Z)} [\mathscr {F}_{ReZG_3}(rs)]^{ \mathscr {F}_{ReZG_3}(rs)}\right]$

## Methodology

In this section, we present the working methodology employed in this study. In “Hyaluronic acid-paclitaxel conjugate”, we compute the entropy measures for hyaluronic acid-paclitaxel conjugate to estimate their physical properties. The following steps are used to compute the entropy measures:

- We convert the hyaluronic acid-paclitaxel conjugate structure into a molecular graph by considering atoms as vertices and chemical bonds as edges.
- We partition the vertices and edges of the graph based on the reverse degree.
- We compute the entropy measures and plot the graphical representation using Maple software.

In “Statistical analysis of entropy measures”, we develop the statistical analysis of entropy measures. The following steps are used for statistical analysis:

- We consider a specific class of drug.
- We take the chemical structures of the drug and convert them into molecular graphs by considering atoms as vertices and chemical bonds as edges.
- We use newGraph software to compute the adjacency matrix from the graph.
- We propose a Maple-based algorithm to compute the entropy measures based on the adjacency matrix.
- We obtain the physical properties of cancer drugs from https://pubchem.ncbi.nlm.nih.gov/ and https://www.chemspider.com/.
- We develop the statistical analysis between entropy measures and the physical properties using Python.

## Hyaluronic acid-paclitaxel conjugate

Cancer is widely recognized as a prominent contributor to global mortality rates, with a persistent upward trend in fatality rates. The primary culprits behind these deaths are breast, stomach, lung, and colon cancers. Despite significant advancements in the field of cancer biology and the development of various therapeutic approaches to combat cancer, there persist challenges in effectively treating both primary and metastatic forms of the disease. Furthermore, it is important to acknowledge the presence of drawbacks in existing anticancer medications, as they often exhibit a lack of specificity and a high level of toxicity, thereby significantly impairing their effectiveness. Significant advancements have been made in the field of molecularly-targeted cancer treatment in recent years.

Hyaluronic acid (HA) is an endogenous compound. The compound in question is a polymer of glycosaminoglycan, consisting of a linear arrangement of D-glucuronic acid and N-acetyl-D-glucosamine units. These units are connected through alternating $\beta$-1,4- and $\beta$-1,3-glycosidic bonds. The primary structure of the disaccharide is considered to be energetically stable^39^. Hyaluronic acid (HA) exhibits considerable potential as a cancer therapeutic agent owing to its distinctive properties, including biodegradability, biocompatibility, non-toxicity, hydrophilicity, and non-immunogenicity. Moreover, the overexpression of HA receptors has been observed on numerous tumor cells, further supporting its promise as a cancer drug. Hyaluronic acid (HA) is currently being recognized as a promising platform for effectively targeting cells that overexpress CD44. The primary objective of utilizing HA in this context is to enhance the efficacy of anticancer treatments^40–42^. Hyaluronic acid (HA) exhibits promising characteristics as a drug carrier and drug-targeting agent. Paclitaxel (PTX) is a pharmacological agent that has demonstrated efficacy in the treatment of various malignancies such as bladder, lung, breast, esophageal, ovarian, and prostate cancers, among others^43^. Although the administration of PTX also faces certain limitations, such as its limited solubility and associated side effects, as well as the excipients commonly employed in its formulation. The initial proposal by Ringsdorf introduced a technique for the synthesis of polymeric macromolecule-drug conjugates. This method was specifically designed to facilitate the targeted delivery of small hydrophobic drug molecules to their intended sites of action^44^. The primary benefits of HA-PTX conjugates include enhanced water solubility and maintained activity. Moreover, these conjugates can be employed as targeted drug delivery systems to enhance the effectiveness of anti-tumor treatment^45–47^.

**Fig. 1:**
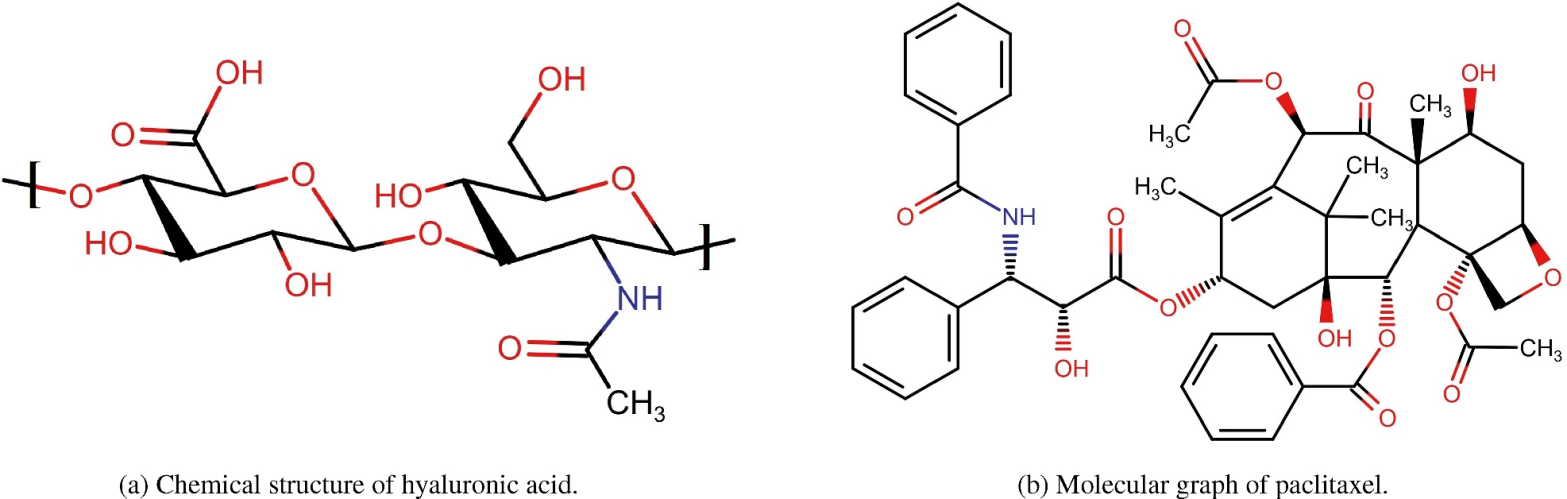
Unit structures of hyaluronic acid and paclitaxel.

**Fig. 2:**
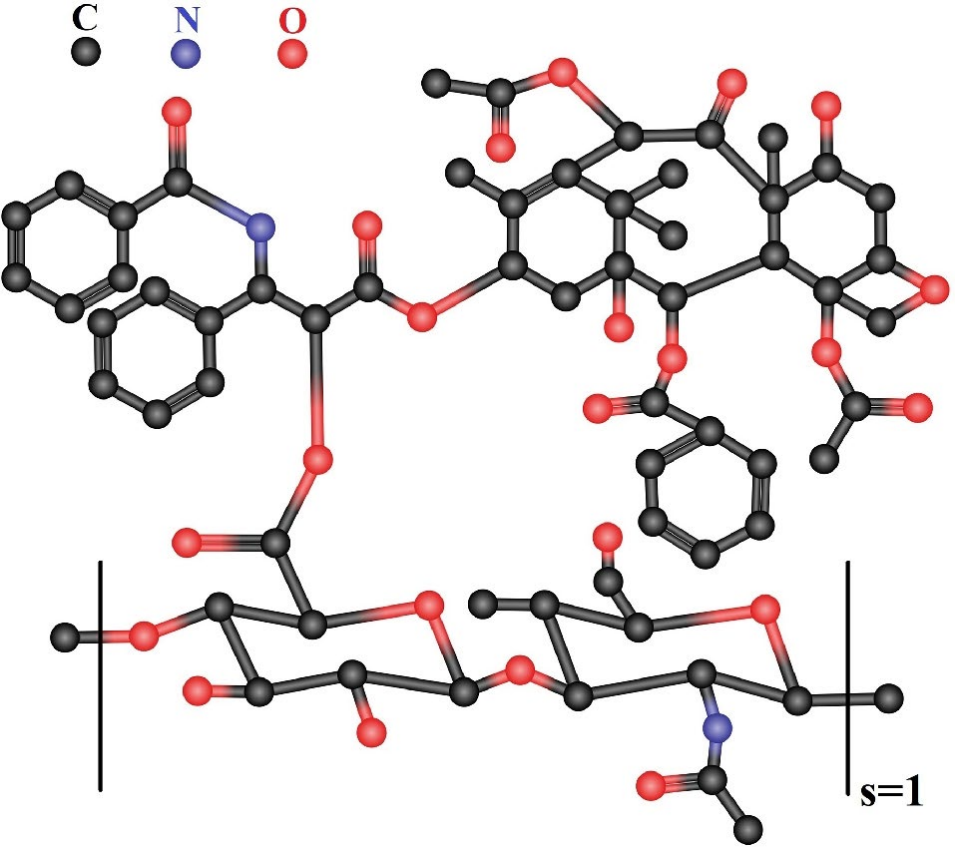
Molecular graph of hyaluronic acid-paclitaxel conjugate for $s=1$.

**Fig. 3:**
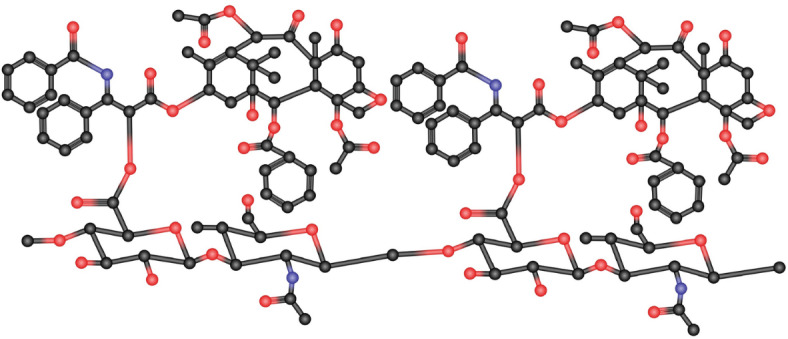
Molecular graph of hyaluronic acid-paclitaxel conjugate for $s=2$.

Figure 1 shows the unit chemical structures of hyaluronic acid and paclitaxel. Figures 2 and 3 illustrates the molecular graph of hyaluronic acid-paclitaxel conjugate for $s=1$ and $s=2$, respectively. If $s<1$, then it means that hyaluronic acid-paclitaxel conjugate has degraded and as result breakdown into two or more parts (glucuronic acid, N-acetylglucosamine and paclitaxel). If $s \ge 1$ then it means that the hyaluronic acid-paclitaxel conjugate polymerization has occurred equal to value of *s*. For example, if $s=2$ then degree of polymerization is 2.

## Main results

Let HA be the molecular structure of hyaluronic acid-paclitaxel conjugate. We partitioned the edges, base on the reverse degree as list in Table 3.

**Table 3:** Edge partition of *HA* based on reverse degree.

Notations for edges	$({\Upsilon }_r, {\Upsilon }_s)$	Frequency of edges
${E_{1}}$	(2, 2)	*s*
${E_{2}}$	(2, 3)	7*s*
${E_{3}}$	(3, 3)	$19s-1$
${E_{4}}$	(2, 4)	3*s*
${E_{5}}$	(3, 4)	$32s-1$
${E_{6}}$	(4, 4)	$13s+1$
${E_{7}}$	(2, 5)	4*s*
${E_{8}}$	(3, 5)	16*s*
${E_{9}}$	(4, 5)	$s+1$

Using Table 3, we will calculate the following entropy measures.

### Atom bond connectivity entropy

$$\begin{aligned} \mathscr {E}_{ABC}(HA)= & log(ABC)-\frac{1}{(ABC)} log [ \prod _{cd\in E_1} \left[ \sqrt{\frac{{\Upsilon }_r+{\Upsilon }_s-2}{{\Upsilon }_r\times {\Upsilon }_s}}\right] ^{\sqrt{\frac{{\Upsilon }_r+{\Upsilon }_s-2}{{\Upsilon }_r\times {\Upsilon }_s}}} \\ & \times \prod _{cd\in E_2} \left[ \sqrt{\frac{{\Upsilon }_r+{\Upsilon }_s-2}{{\Upsilon }_r \times {\Upsilon }_s}}\right] ^{\sqrt{\frac{{\Upsilon }_r+{\Upsilon }_s-2}{{\Upsilon }_r\times {\Upsilon }_s}} } \\\times & \prod _{cd\in E_3} \left[ \sqrt{\frac{{\Upsilon }_r+{\Upsilon }_s-2}{{\Upsilon }_r\times {\Upsilon }_s}}\right] ^{\sqrt{\frac{{\Upsilon }_r+{\Upsilon }_s-2}{{\Upsilon }_r\times {\Upsilon }_s}}}\times \prod _{cd\in E_4} \left[ \sqrt{\frac{{\Upsilon }_r+{\Upsilon }_s-2}{{\Upsilon }_r\times {\Upsilon }_s}}\right] ^{\sqrt{\frac{{\Upsilon }_r+{\Upsilon }_s-2}{{\Upsilon }_r\times {\Upsilon }_s}}} \\ & \times \prod _{cd\in E_5} \left[ \sqrt{\frac{{\Upsilon }_r+{\Upsilon }_s-2}{{\Upsilon }_r\times {\Upsilon }_s}}\right] ^{\sqrt{\frac{{\Upsilon }_r+{\Upsilon }_s-2}{{\Upsilon }_r\times {\Upsilon }_s}}}\\\times & \prod _{cd\in E_6} \left[ \sqrt{\frac{{\Upsilon }_r+{\Upsilon }_s-2}{{\Upsilon }_r\times {\Upsilon }_s}}\right] ^{\sqrt{\frac{{\Upsilon }_r+{\Upsilon }_s-2}{{\Upsilon }_r\times {\Upsilon }_s}}} \times \prod _{cd\in E_7} \left[ \sqrt{\frac{{\Upsilon }_r+{\Upsilon }_s-2}{{\Upsilon }_r\times {\Upsilon }_s}}\right] ^{\sqrt{\frac{{\Upsilon }_r+{\Upsilon }_s-2}{{\Upsilon }_r\times {\Upsilon }_s}}} \\ & \times \prod _{cd\in E_8} \left[ \sqrt{\frac{{\Upsilon }_r+{\Upsilon }_s-2}{{\Upsilon }_r \times {\Upsilon }_s}}\right] ^{\sqrt{\frac{{\Upsilon }_r+{\Upsilon }_s-2}{{\Upsilon }_r\times {\Upsilon }_s}}} \\\times & \prod _{cd\in E_9} \left[ \sqrt{\frac{{\Upsilon }_r+{\Upsilon }_s-2}{{\Upsilon }_r\times {\Upsilon }_s}}\right] ^{\sqrt{\frac{{\Upsilon }_r+{\Upsilon }_s-2}{{\Upsilon }_r\times {\Upsilon }_s}}} ], \\ \\ \mathscr {E}_{ABC}(HA)= & log \left[ 62.6009s-0.1082\right] - \frac{1}{\left[ 62.6009s-0.1082\right] } [ log \left[ (s)(\frac{1}{\sqrt{2}})^{\frac{1}{\sqrt{2}}}\right] + log \left[ (7s)(\sqrt{\frac{3}{6}})^{\sqrt{\frac{3}{6}}}\right] \\+ & log\left[ (19s-1)(\sqrt{\frac{4}{9}})^{\sqrt{\frac{4}{9}}}\right] +log\left[ (3s)(\frac{1}{\sqrt{2}})^{\frac{1}{\sqrt{2}}}\right] \\ & + log\left[ (32s-1)(\sqrt{\frac{5}{12}})^{\sqrt{\frac{5}{12}}}\right] +log\left[ (13s+1)(\sqrt{\frac{6}{16}})^{\sqrt{\frac{6}{16}}}\right] \\+ & log\left[ (4s)(\frac{1}{\sqrt{2}})^{\frac{1}{\sqrt{2}}}\right] +log\left[ (16s)(\sqrt{\frac{6}{15}})^{\sqrt{\frac{6}{15}}}\right] +log\left[ (s+1)(\sqrt{\frac{7}{20}})^{\sqrt{\frac{7}{20}}}\right] ]. \end{aligned}$$

### Geometric arithmetic entropy

$$\begin{aligned} \mathscr {E}_{GA}(HA)= & log(GA)-\frac{1}{(GA)}log[\prod _{cd\in E_1} \left[ \frac{2\sqrt{{{\Upsilon }_r\times {\Upsilon }_s}}}{{{\Upsilon }_r+ {\Upsilon }_s}}\right] ^{\left[ \frac{2\sqrt{{{\Upsilon }_r\times {\Upsilon }_s}}}{{{\Upsilon }_r+ {\Upsilon }_s}}\right] } \times \prod _{cd\in E_2} \left[ \frac{2\sqrt{{{\Upsilon }_r\times {\Upsilon }_s}}}{{{\Upsilon }_r+ {\Upsilon }_s}}\right] ^{\left[ \frac{2\sqrt{{{\Upsilon }_r\times {\Upsilon }_s}}}{{{\Upsilon }_r+ {\Upsilon }_s}}\right] } \\\times & \prod _{cd\in E_3} \left[ \frac{2\sqrt{{{\Upsilon }_r\times {\Upsilon }_s}}}{{{\Upsilon }_r+ {\Upsilon }_s}}\right] ^{\left[ \frac{2\sqrt{{{\Upsilon }_r\times {\Upsilon }_s}}}{{{\Upsilon }_r+ {\Upsilon }_s}}\right] } \times \prod _{cd\in E_4} \left[ \frac{2\sqrt{{{\Upsilon }_r\times {\Upsilon }_s}}}{{{\Upsilon }_r+ {\Upsilon }_s}}\right] ^{\left[ \frac{2\sqrt{{{\Upsilon }_r\times {\Upsilon }_s}}}{{{\Upsilon }_r+ {\Upsilon }_s}}\right] } \times \prod _{cd\in E_5} \left[ \frac{2\sqrt{{{\Upsilon }_r\times {\Upsilon }_s}}}{{{\Upsilon }_r+ {\Upsilon }_s}}\right] ^{\left[ \frac{2\sqrt{{{\Upsilon }_r\times {\Upsilon }_s}}}{{{\Upsilon }_r+ {\Upsilon }_s}}\right] }\\\times & \prod _{cd\in E_6} \left[ \frac{2\sqrt{{{\Upsilon }_r\times {\Upsilon }_s}}}{{{\Upsilon }_r+ {\Upsilon }_s}}\right] ^{\left[ \frac{2\sqrt{{{\Upsilon }_r\times {\Upsilon }_s}}}{{{\Upsilon }_r+ {\Upsilon }_s}}\right] }\times \prod _{cd\in E_7} \left[ \frac{2\sqrt{{{\Upsilon }_r\times {\Upsilon }_s}}}{{{\Upsilon }_r+ {\Upsilon }_s}}\right] ^{\left[ \frac{2\sqrt{{{\Upsilon }_r\times {\Upsilon }_s}}}{{{\Upsilon }_r+ {\Upsilon }_s}}\right] }\\\times & \prod _{cd\in E_8} \left[ \frac{2\sqrt{{{\Upsilon }_r\times {\Upsilon }_s}}}{{{\Upsilon }_r+ {\Upsilon }_s}}\right] ^{\left[ \frac{2\sqrt{{{\Upsilon }_r\times {\Upsilon }_s}}}{{{\Upsilon }_r+ {\Upsilon }_s}}\right] }\times \prod _{cd\in E_9} \left[ \frac{2\sqrt{{{\Upsilon }_r\times {\Upsilon }_s}}}{{{\Upsilon }_r+ {\Upsilon }_s}}\right] ^{\left[ \frac{2\sqrt{{{\Upsilon }_r\times {\Upsilon }_s}}}{{{\Upsilon }_r+ {\Upsilon }_s}}\right] }]\\ \\ \mathscr {E}_{GA}(HA)= & log\left[ 93.9919s+1.9836\right] - \frac{1}{93.9919s+1.9836}[log\left[ (s)\left[ \frac{2\sqrt{4}}{4}\right] ^{(\frac{2\sqrt{4}}{4})}\right] \\+ & log \left[ (7s)\left[ \frac{2\sqrt{6}}{5}\right] ^{(\frac{2\sqrt{6}}{5})}\right] + log\left[ (19s-1)\left[ \frac{2\sqrt{9}}{6}\right] ^{(\frac{2\sqrt{9}}{6})}\right] +log\left[ (3s)\left[ \frac{2\sqrt{8}}{6}\right] ^{(\frac{2\sqrt{8}}{6})}\right] \\+ & log\left[ (32s-1)\left[ \frac{2\sqrt{12}}{7}\right] ^{(\frac{2\sqrt{12}}{7})}\right] +log\left[ (13s+1)\left[ \frac{2\sqrt{16}}{8}\right] ^{(\frac{2\sqrt{16}}{8})}\right] +log\left[ (4s)\left[ \frac{2\sqrt{10}}{7}\right] ^{(\frac{2\sqrt{10}}{7})}\right] \\+ & log\left[ (16s)\left[ \frac{2\sqrt{15}}{8}\right] ^{(\frac{2\sqrt{15}}{8})}\right] +log\left[ (s+1)\left[ \frac{2\sqrt{20}}{9}\right] ^{(\frac{2\sqrt{20}}{9})}\right] ]. \end{aligned}$$

### First zagreb entropy

$$\begin{aligned} \mathscr {E}_{M_1}(HA)= & log(M_1)-\frac{1}{(M_1)}log[\left[ \prod _{cd\in E_1} [{\Upsilon }_r+{\Upsilon }_s]^{({\Upsilon }_r+{\Upsilon }_s)}\right] \times \left[ \prod _{cd\in E_2} [{\Upsilon }_r+{\Upsilon }_s]^{({\Upsilon }_r+{\Upsilon }_s)}\right] \\ & \times \left[ \prod _{cd\in E_3} [{\Upsilon }_r+{\Upsilon }_s]^{({\Upsilon }_r+{\Upsilon }_s)}\right] \\\times & \left[ \prod _{cd\in E_4} [{\Upsilon }_r+{\Upsilon }_s]^{({\Upsilon }_r+{\Upsilon }_s)}\right] \times \left[ \prod _{cd\in E_5} [{\Upsilon }_r+{\Upsilon }_s]^{({\Upsilon }_r+{\Upsilon }_s)}\right] \times \left[ \prod _{cd\in E_6} [{\Upsilon }_r+{\Upsilon }_s]^{({\Upsilon }_r+{\Upsilon }_s)}\right] \\\times & \left[ \prod _{cd\in E_7} [{\Upsilon }_r+{\Upsilon }_s]^{({\Upsilon }_r+{\Upsilon }_s)}\right] \times \left[ \prod _{cd\in E_8} [{\Upsilon }_r+{\Upsilon }_s]^{({\Upsilon }_r+{\Upsilon }_s)}\right] \times \left[ \prod _{cd\in E_9} [{\Upsilon }_r+{\Upsilon }_s]^{({\Upsilon }_r+{\Upsilon }_s)}\right] ] \\ \mathscr {E}_{M_1}(HA)= & log(664s+4)-\frac{1}{664s+4}[log\left[ (s)\times (4)^{4}\right] +log\left[ (7s)\times (5)^{5}\right] +log\left[ (19s-1)\times (6)^{6}\right] \\+ & log\left[ (3s)\times (6)^{6}\right] +log\left[ (32s-1)\times (7)^{7}\right] +log\left[ (13s+1)\times (8)^{8}\right] \\+ & log\left[ (4s)\times (7)^{7}\right] +log\left[ (16s)\times (8)^{8}\right] + log\left[ (s+1)\times (9)^{9}\right] ]. \end{aligned}$$

### Second zagreb entropy

$$\begin{aligned} \mathscr {E}_{M_2}(HA)= & log(M_2)-\frac{1}{(M_2)}log[\left[ \prod _{cd\in E_1} [{\Upsilon }_r\times {\Upsilon }_s]^{({\Upsilon }_r\times {\Upsilon }_s)}\right] \times \left[ \prod _{cd\in E_2} [{\Upsilon }_r\times {\Upsilon }_s]^{({\Upsilon }_r\times {\Upsilon }_s)}\right] \\ & \times \left[ \prod _{cd\in E_3} [{\Upsilon }_r\times {\Upsilon }_s]^{({\Upsilon }_r\times {\Upsilon }_s)}\right] \\\times & \left[ \prod _{cd\in E_4} [{\Upsilon }_r\times {\Upsilon }_s]^{({\Upsilon }_r\times {\Upsilon }_s)}\right] \times \left[ \prod _{cd\in E_5} [{\Upsilon }_r\times {\Upsilon }_s]^{({\Upsilon }_r\times {\Upsilon }_s)}\right] \times \left[ \prod _{cd\in E_6} [{\Upsilon }_r\times {\Upsilon }_s]^{({\Upsilon }_r\times {\Upsilon }_s)}\right] \\\times & \left[ \prod _{cd\in E_7} [{\Upsilon }_r\times {\Upsilon }_s]^{({\Upsilon }_r\times {\Upsilon }_s)}\right] \times \left[ \prod _{cd\in E_8} [{\Upsilon }_r\times {\Upsilon }_s]^{({\Upsilon }_r\times {\Upsilon }_s)}\right] \times \left[ \prod _{cd\in E_9} [{\Upsilon }_r\times {\Upsilon }_s]^{({\Upsilon }_r\times {\Upsilon }_s)}\right] ] \\ \mathscr {E}_{M_2}(HA)= & log(113s+15)-\frac{1}{113s+15}[log\left[ (s)\times (4)^{4}\right] +log\left[ (7s)\times (6)^{6}\right] + log\left[ (19s-1)\times (9)^{9}\right] \\+ & log\left[ (3s)\times (8)^{8}\right] +log\left[ (32s-1)\times (12)^{12}\right] + log\left[ (13s+1)\times (16)^{16}\right] \\+ & log\left[ (4s)\times (10)^{10}\right] +log\left[ (16s)\times (15)^{15}\right] + log\left[ (s+1)\times (20)^{20}\right] ]. \end{aligned}$$

### Hyper first zagreb entropy

$$\begin{aligned} \mathscr {E}_{HM_1}(HA)= & log(HM_1)-\frac{1}{(HM_1)}log[\left[ \prod _{cd\in E_1} [({\Upsilon }_r+{\Upsilon }_s)^2]^{({\Upsilon }_r+{\Upsilon }_s)^2}\right] \times \left[ \prod _{cd\in E_2} [({\Upsilon }_r+{\Upsilon }_s)^2]^{({\Upsilon }_r+{\Upsilon }_s)^2}\right] \\ & \times \left[ \prod _{cd\in E_3} [({\Upsilon }_r+{\Upsilon }_s)^2]^{({\Upsilon }_r+{\Upsilon }_s)^2}\right] \\\times & \left[ \prod _{cd\in E_4} [({\Upsilon }_r+{\Upsilon }_s)^2]^{({\Upsilon }_r+{\Upsilon }_s)^2}\right] \times \left[ \prod _{cd\in E_5} [({\Upsilon }_r+{\Upsilon }_s)^2]^{({\Upsilon }_r+{\Upsilon }_s)^2}\right] \times \left[ \prod _{cd\in E_6} [({\Upsilon }_r+{\Upsilon }_s)^2]^{({\Upsilon }_r+{\Upsilon }_s)^2}\right] \\\times & \left[ \prod _{cd\in E_7} [({\Upsilon }_r+{\Upsilon }_s)^2]^{({\Upsilon }_r+{\Upsilon }_s)^2}\right] \times \left[ \prod _{cd\in E_8} [({\Upsilon }_r+{\Upsilon }_s)^2]^{({\Upsilon }_r+{\Upsilon }_s)^2}\right] \times \left[ \prod _{cd\in E_9} [({\Upsilon }_r+{\Upsilon }_s)^2]^{({\Upsilon }_r+{\Upsilon }_s)^2}\right] ]\\ \mathscr {E}_{HM_1}(HA)= & log(4684s+60)-\frac{1}{4684s+60}[log\left[ (s)\times (16)^{16}\right] +log\left[ (7s)\times (25)^{25}\right] log\left[ (19s-1)\times (36)^{36}\right] \\+ & log\left[ (3s)\times (36)^{36}\right] +log\left[ (32s-1)\times (49)^{49}\right] + log\left[ (13s+1)\times (64)^{64}\right] \\+ & log\left[ (4s)\times (49)^{49}\right] +log\left[ (16s)\times (64)^{64}\right] + log\left[ (s+1)\times (81)^{81}\right] ]. \end{aligned}$$

### Forgotten entropy

$$\begin{aligned} \mathscr {E}_{F}(HA)= & log(F)-\frac{1}{(F)}log[\left[ \prod _{cd\in E_1} \left[ ({\Upsilon }_r)^2+({\Upsilon }_s)^2\right] ^{({\Upsilon }_r)^2+({\Upsilon }_s)^2}\right] \\\times & \left[ \prod _{cd\in E_2} \left[ ({\Upsilon }_r)^2+({\Upsilon }_s)^2\right] ^{({\Upsilon }_r)^2+({\Upsilon }_s)^2}\right] \times \left[ \prod _{cd\in E_3} \left[ ({\Upsilon }_r)^2+({\Upsilon }_s)^2\right] ^{({\Upsilon }_r)^2+({\Upsilon }_s)^2}\right] \\\times & \left[ \prod _{cd\in E_4} \left[ ({\Upsilon }_r)^2+({\Upsilon }_s)^2\right] ^{({\Upsilon }_r)^2+({\Upsilon }_s)^2}\right] \times \left[ \prod _{cd\in E_5} \left[ ({\Upsilon }_r)^2+({\Upsilon }_s)^2\right] ^{({\Upsilon }_r)^2+({\Upsilon }_s)^2}\right] \\\times & \left[ \prod _{cd\in E_6} \left[ ({\Upsilon }_r)^2+({\Upsilon }_s)^2\right] ^{({\Upsilon }_r)^2+({\Upsilon }_s)^2}\right] \times \left[ \prod _{cd\in E_7} \left[ ({\Upsilon }_r)^2+({\Upsilon }_s)^2\right] ^{({\Upsilon }_r)^2+({\Upsilon }_s)^2}\right] \\\times & \left[ \prod _{cd\in E_8} \left[ ({\Upsilon }_r)^2+({\Upsilon }_s)^2\right] ^{({\Upsilon }_r)^2+({\Upsilon }_s)^2}\right] \times \left[ \prod _{cd\in E_9} \left[ ({\Upsilon }_r)^2+({\Upsilon }_s)^2\right] ^{({\Upsilon }_r)^2+({\Upsilon }_s)^2}\right] ]\\ \mathscr {E}_{F}(HA)= & log(2418s+30)-\frac{1}{2418s+30}[log\left[ (s)\times (8)^{8}\right] +log\left[ (7s)\times (13)^{13}\right] \\+ & log\left[ (19s-1)\times (18)^{18}\right] +log\left[ (3s)\times (20)^{20}\right] +log\left[ (32s-1)\times (25)^{25}\right] \\+ & log\left[ (13s+1)\times (32)^{32}\right] +log\left[ (4s)\times (29)^{29}\right] +log\left[ (16s)\times (34)^{34}\right] \\+ & log\left[ (s+1)\times (41)^{41}\right] ]. \end{aligned}$$

**Fig. 4:**
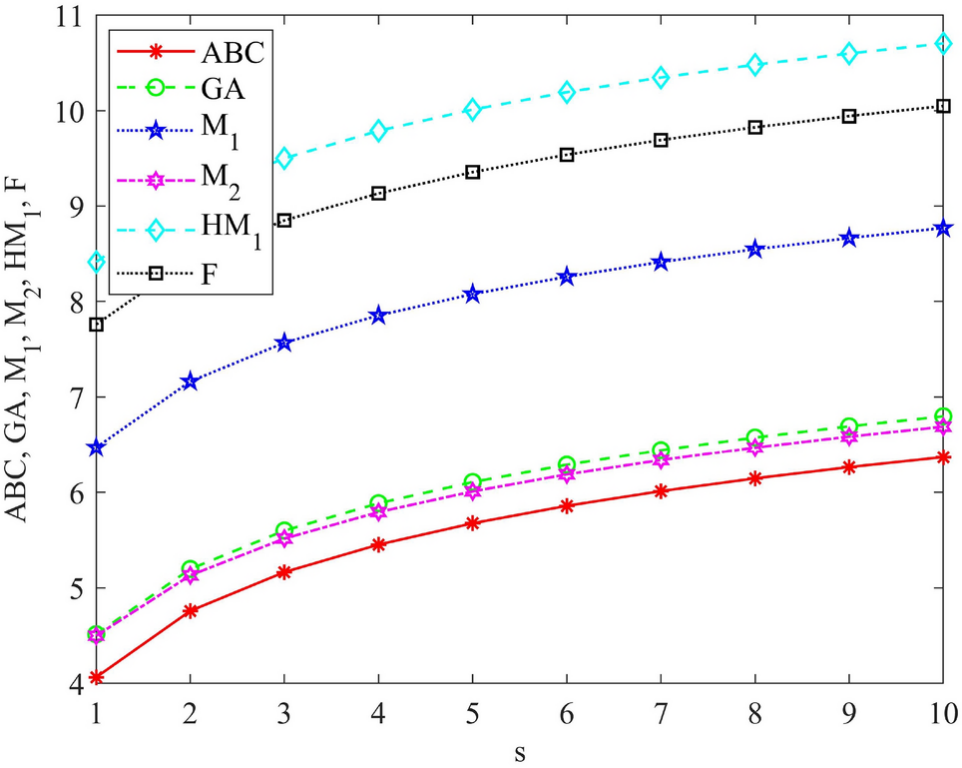
Graphical representation of $\mathscr {E}_{ABC}$, $\mathscr {E}_{GA}$, $\mathscr {E}_{M_1}$, $\mathscr {E}_{M_2}$, $\mathscr {E}_{HM_1}$ and $\mathscr {E}_{F}$.

Graphical representations and numerical values of entropy measures are shown in Fig. 4 and Table 4.

**Table 4:** Numerical representation of the reverse entropy measures.

[*s*]	$\mathscr {E}_{ABC}$	$\mathscr {E}_{GA}$	$\mathscr {E}_{M_1}$	$\mathscr {E}_{M_2}$	$\mathscr {E}_{HM_1}$	$\mathscr {E}_{F}$
[1]	4.0652	4.5147	6.4724	4.4986	8.4133	7.7584
[2]	4.7592	5.1975	7.1625	5.1314	9.1001	8.4454
[3]	5.165	5.5994	7.567	5.5159	9.5034	8.8488
[4]	5.4528	5.8854	7.8542	5.7929	9.7901	9.1355
[5]	5.676	6.1075	8.077	6.0096	10.0126	9.358
[6]	5.8584	6.2891	8.2591	6.1876	10.1945	9.5399
[7]	6.0126	6.4427	8.4131	6.3387	10.3483	9.6938
[8]	6.1461	6.5759	8.5466	6.4699	10.4816	9.8271
[9]	6.2639	6.6934	8.6643	6.5859	10.5992	9.9447
[10]	6.3693	6.7985	8.7696	6.6898	10.7044	10.0499

## Statistical analysis of entropy measures

       Quantitative structure-property relationship (QSPR) studies have emerged as a vital tool in predicting physical properties of molecules using topological indices. These indices, derived from molecular graphs, encode structural information that correlates with physical properties of drugs.

**Fig. 5:**
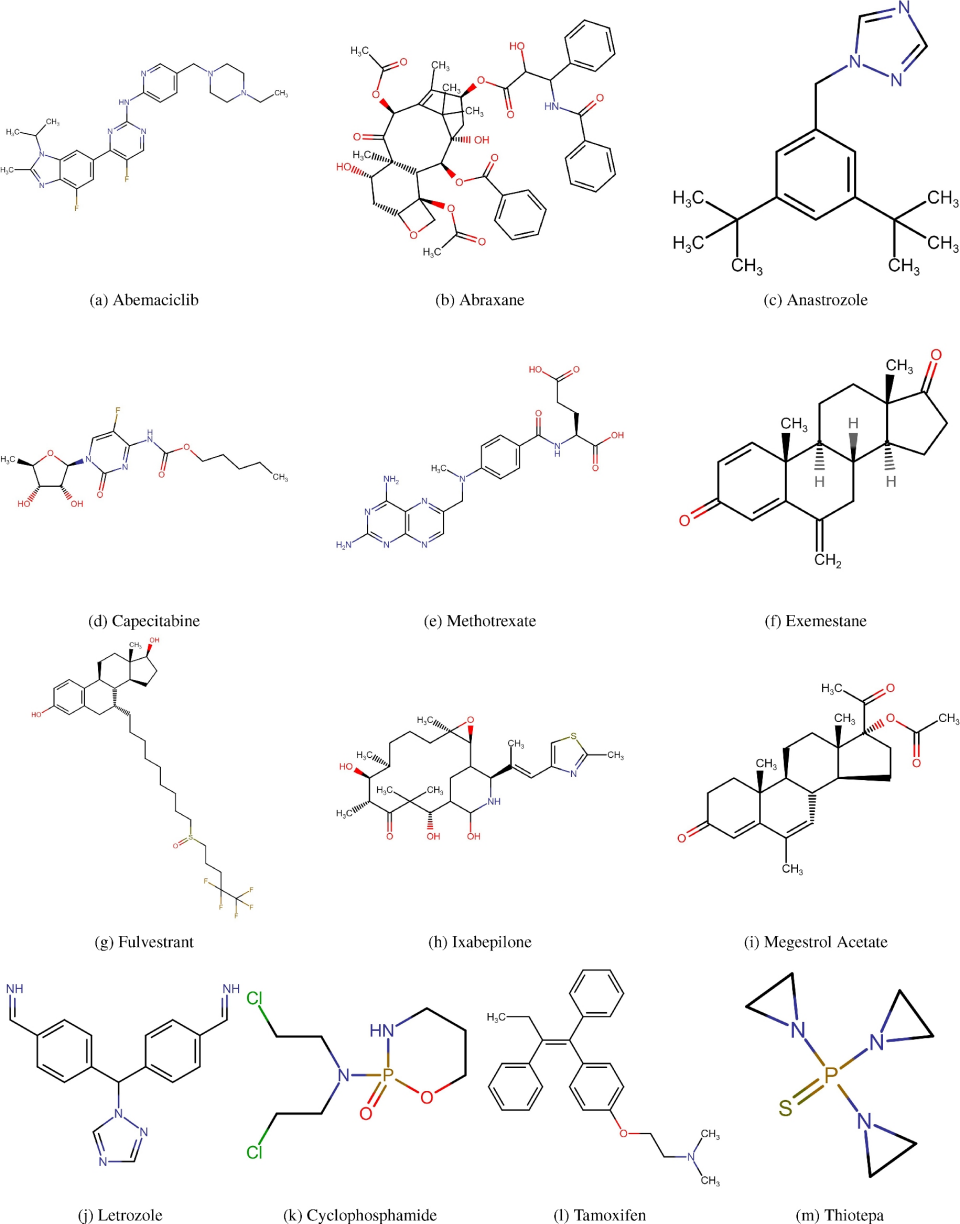
Chemical structures of cancer drugs.

**Fig. 6:**
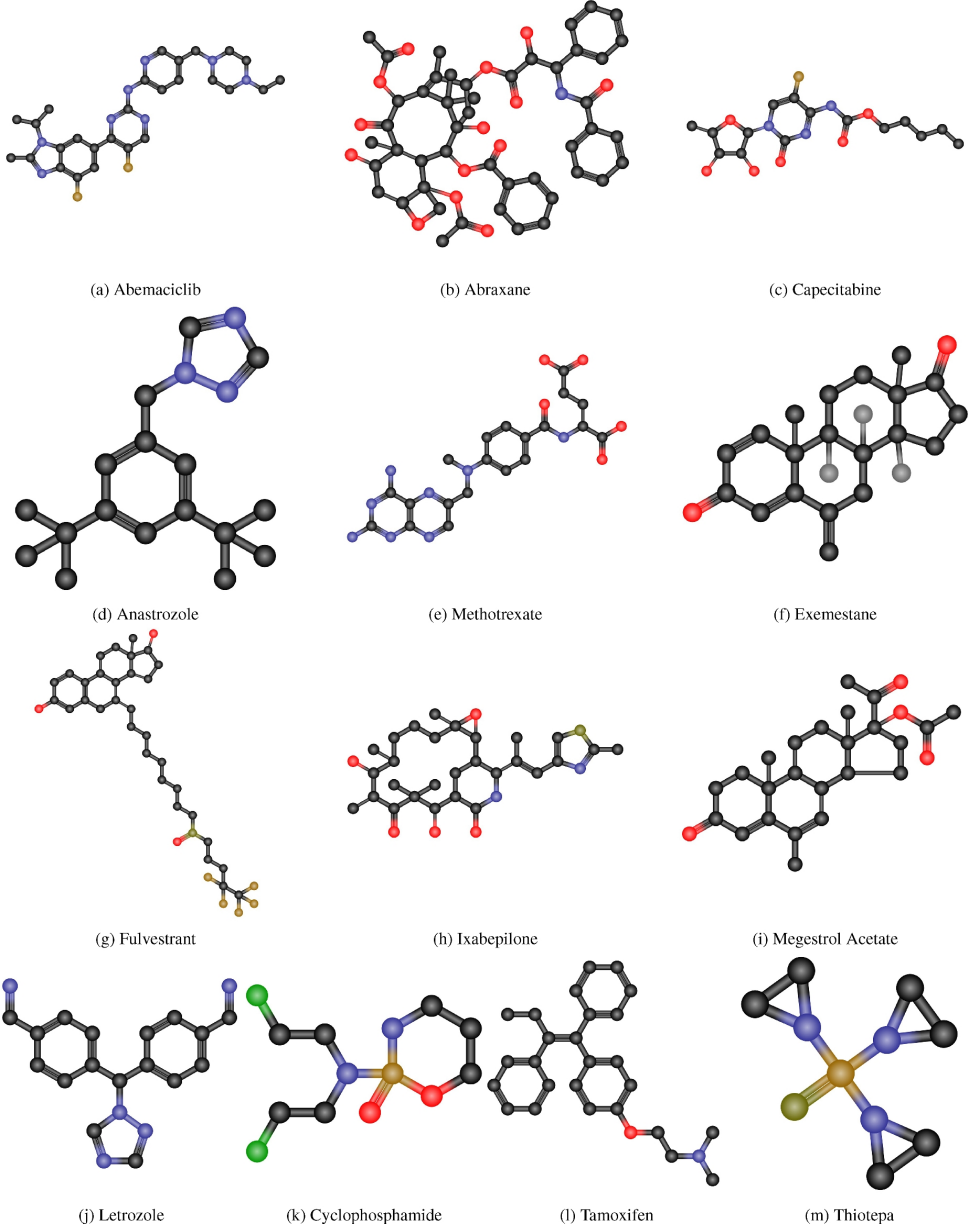
Molecular graphs of cancer drugs.

The QSPR between the physical characteristics of cancer medications and their entropy measures is being developed in this area. The cancer medications shown in Fig. 5. To compute the entropy measures, first we convert the chemical structure of drugs into molecular graph given in Fig. 6. The entropy measures value given in Table 5 and the physical properties in Table 6.

**Table 5:** Entropy measures values for cancer drugs.

Drugs	AZI	$M_1$	$M_2$	$^mM_2$	*H*	$ReZG_3$	*SDD*	*I*	*F*
Abemaciclib	98.1406	58	63	3.1111	6	298	30	13.6667	140
Abraxane	592.718	361	458	13.1181	28.2357	2580	165.333	84.5976	1023
Anastrozole	139.701	94	108	3.6944	7.819	558	47.8333	21.3643	258
Capecitabine	213.219	128	149	6	11.8333	760	63.3333	30.0833	330
Cyclophosphamide	112.194	64	72	3.4167	6.4857	368	32.6667	14.9143	166
Exemestane	240.867	156	208	4.9861	10.5714	1308	74.75	34.8452	500
Fulvestrant	340.536	212	251	8.6181	18.0548	1346	105	48.8952	584
Ixabepilone	273.146	186	220	7.4444	15.1571	1188	95	42.0095	524
Letrozole	202.172	112	131	5.0833	10.6333	646	51	27.2333	276
Megestrol Acetate	253.422	164	209	5.8125	12.2262	1218	78.1667	37.3905	488
Methotrexate	279.359	172	200	7.6111	15.5	1014	84.3333	40.4167	444
Tamoxifen	244.313	138	158	6.4444	13.4	768	65	33.3667	336
Thiotepa	115.842	68	88	2.25	5.1571	500	29.5	16.1429	194

**Table 6:** The physical properties of cancer drugs.

Drugs	BP	EoV	FP	MR	MV	P	MW	MM	PSA	HAC	C
Abemaciclib	689.3	101	370.7	140.4	382.3	55.7	506.6	506.27	75	37	738
Abraxane	957.1	146	532.6	219.3	610.6	86.9	853.9	853.3	221	62	1790
Anastrozole	469.7	73.2	237.9	90	270.3	35.7	293.4	293.16	78.3	22	456
Capecitabine	517.0	81.2	87	82.3	240.5	32.6	359.4	359.1	121	25	582
Cyclophosphamide	336.1	57.9	157.1	58.1	195.7	23	261.1	260.02	41.6	14	212
Exemestane	453.7	71.3	169	85.8	260.6	34	296.4	296.17	34.1	22	653
Fulvestrant	674.8	104.1	361.9	154	505.1	61.1	606.8	606.31	76.7	41	854
Ixabepilone	697.8	107.3	375.8	140.1	451.6	55.5	506.7	506.2	140	35	817
Letrozole	563.5	84.7	294.6	87.1	234.5	34.5	285.3	285.1	78.3	22	420
Megestrol Acetate	507.1	77.7	77.7	106.4	333.4	42.2	384.5	384.23	60.4	28	821
Methotrexate	561.3	84.1	284.15	119	295.7	47.2	454.4	454.17	211	33	704
Tamoxifen	482.3	74.7	140	118.9	118.9	47.1	371.5	371.2	12.5	28	463
Thiotepa	270.2	50.8	117.2	49.1	125.8	19.5	189.2	189.04	41.1	11	194

To propose the relationship between entropy measures and the physical properties of cancer drug, multiple linear (MLR), Ridge (RR), Lasso (LR), ElasticNet (ENR) and Support Vector regression (SVR) are used. We calculate the only correlation coefficient, coefficient of determination and mean squared error to decide that which model predict the desire physical property listed in Tables 7, 8, 9, 10, 11, 12, 13, 14, 15, 16 and 17.

**Table 7:** Regression model for BP.

Model	Pearson R	Coef. of determination	Mean squared error
Multiple linear regression	$-0.477$	0.228	100534.7691
Ridge regression	0.999	0.997	3402.2063
Lasso regression	0.999	0.998	3349.7946
ElasticNet regression	0.999	0.997	3517.0989
Support vector regression	0.993	0.986	8921.9773

**Table 8:** Regression model for EoV.

Model	Pearson R	Coef. of determination	Mean squared error
Multiple linear regression	$-0.559$	0.313	1892.3409
Ridge regression	0.999	0.998	48.2737
Lasso regression	0.999	0.998	49.7108
ElasticNet regression	0.999	0.998	51.3297
Support vector regression	0.992	0.984	139.9596

**Table 9:** Regression model for FP.

Model	Pearson R	Coef. of determination	Mean squared error
Multiple linear regression	$-0.346$	0.120	92689.8022
Ridge regression	0.965	0.931	15647.8733
Lasso regression	0.968	0.938	15416.2368
ElasticNet regression	0.966	0.934	15801.9674
Support vector regression	0.908	0.824	20067.9223

**Table 10:** Regression model for MR.

Model	Pearson R	Coef. of determination	Mean squared error
Multiple linear regression	$-0.684$	0.467	3201.1490
Ridge regression	0.902	0.813	253.6365
Lasso regression	0.908	0.824	245.7076
ElasticNet regression	0.908	0.825	250.6917
Support vector regression	0.899	0.808	1256.6219

**Table 11:** Regression model for MV.

Model	Pearson R	Coef. of determination	Mean squared error
Multiple linear regression	0.219	0.048	69903.6016
Ridge regression	0.707	0.500	11182.2656
Lasso regression	1.000	1.000	10708.8748
ElasticNet regression	0.690	0.476	11344.4313
Support vector regression	0.704	0.495	13154.8234

**Table 12:** Regression model for P.

Model	Pearson R	Coef. of determination	Mean squared error
Multiple linear regression	$-0.678$	0.459	498.1708
Ridge regression	0.904	0.817	39.9896
Lasso regression	0.910	0.828	39.0514
ElasticNet regression	0.910	0.829	39.6940
Support vector regression	0.890	0.791	193.7721

**Table 13:** Regression model for MW.

Model	Pearson R	Coef. of determination	Mean squared error
Multiple linear regression	$-0.417$	0.174	63525.0926
Ridge regression	1.000	1.000	885.8908
Lasso regression	0.915	0.838	1903.2440
ElasticNet regression	1.000	0.999	897.8231
Support vector regression	1.000	0.999	12622.7928

**Table 14:** Regression model for MM.

Model	Pearson R	Coef. of determination	Mean squared error
Multiple linear regression	$-0.416$	0.173	63042.1280
Ridge regression	1.000	1.000	887.2696
Lasso regression	0.917	0.840	1898.1879
ElasticNet regression	1.000	0.999	899.1999
Support vector regression	1.000	0.999	12267.7786

**Table 15:** Regression model for TPSA.

Model	Pearson R	Coef. of determination	Mean squared error
Multiple linear regression	0.998	0.996	8327.3621
Ridge regression	0.681	0.464	3427.4200
Lasso regression	0.681	0.463	3450.3741
ElasticNet regression	0.711	0.506	3384.5901
Support vector regression	0.733	0.537	1560.9485

**Table 16:** Regression model for HAC.

Model	Pearson R	Coef. of determination	Mean squared error
Multiple linear regression	$-0.338$	0.114	200.2285
Ridge regression	0.997	0.995	8.9858
Lasso regression	0.998	0.996	9.0149
ElasticNet regression	0.998	0.996	9.2303
Support vector regression	0.998	0.995	65.6067

**Table 17:** Regression model for C.

Model	Pearson R	Coef. of determination	Mean squared error
Multiple linear regression	0.799	0.638	37851.3148
Ridge regression	0.380	0.145	20700.5452
Lasso regression	0.996	0.993	11751.3839
ElasticNet regression	0.355	0.126	21292.8623
Support vector regression	0.475	0.225	26543.2514

By using Multiple Linear, Ridge, Lasso, Elastic Net, and Support Vector regression, we proposed the models against each physical property for boiling point (BP), enthalpy of vaporization (EoV), flash point (FP), molar refractivity (MR), molar volume (MV), polarization (P), molecular weight (MW), monoisotopic mass (MM), topological polar surface area (TPSA) and complexity (C). Here we are writing only those model that shows the most significant relationship.

4 $$\begin{aligned} \text {Lasso Regression : BP}&= 550.1200 + (165.7870) E_{ReZG_3}, \end{aligned}$$

5 $$\begin{aligned} \text {Ridge Regression : EoV}&= 86.0600 + (2.1374) E_{R_1} + (0.2140) E_{R_{-1}} \nonumber \\&+ (1.1891) E_{R_{1/2}} + (0.9586) E_{R_{-1/2}}+ (0.9586) E_J \nonumber \\&+ (1.0045) E_{GA} + (0.8850) E_{ABC} + (1.3779) E_{M_1} + (2.1374) E_{M_2}\nonumber \\&+ (2.9152) E_F + (2.3871) E_{HM} \nonumber \\&+ (1.3474) E_{AZI} + (0.6050) E_{ReZG_1} + (1.1075) E_{ReZG_2} + (3.8297) E_{ReZG_3}, \end{aligned}$$

6 $$\begin{aligned} \text {Lasso Regression : FP}&= 261.7250 - (28.6916) E_{R_{-1}} - (4.4866) E_{R_{-1/2}} - (110.9448) E_{GA} \nonumber \\&- (60.1245) E_{ABC} - (42.4353) E_{ReZG_1} + (337.8187) E_{ReZG_3}, \end{aligned}$$

7 $$\begin{aligned} \text {Lasso Regression : MR}&= 108.4800 + (41.7586) E_{ReZG_3}, \end{aligned}$$

8 $$\begin{aligned} \text {Lasso Regression : MV}&= 319.0400 + (-360.6798) E_{R_{-1}} + (479.8992) E_{ReZG_3}, \end{aligned}$$

9 $$\begin{aligned} \text {Lasso Regression : P}&= 43.000 + (15.9447) E_{ReZG_3} , \end{aligned}$$

10 $$\begin{aligned} \text {Ridge Regression : MW}&= 410.6550 + (17.6518) E_{R_1} + (-10.7141) E_{R_{-1}} + (9.0215) E_{R_{1/2}} \nonumber \\&+ (5.4112) E_{R_{-1/2}} + (5.4112) E_J \nonumber \\&+ (7.7493) E_{GA} + (6.4299) E_{ABC} + (10.7622) E_{M_1} \nonumber \\&+ (17.6518) E_{M_2} + (23.6332) E_F + (20.3607) E_{HM} \nonumber \\&+ (4.2815) E_{AZI} + (1.5745) E_{ReZG_1} + (7.6497) E_{ReZG_2} + (36.4554) E_{ReZG_3}, \end{aligned}$$

11 $$\begin{aligned} \text {Ridge Regression : MM}&= 410.2570 + (17.6365) E_{R_1} + (-10.6745) E_{R_{-1}} \nonumber \\&+ (9.0273) E_{R_{1/2}} + (5.4242) E_{R_{-1/2}} + (5.4242) E_J\nonumber \\&+ (7.7591) E_{GA} + (6.4450) E_{ABC} + (10.7603) E_{M_1} \nonumber \\&+ (17.6365) E_{M_2} + (23.5873) E_F + (20.3302)E_{HM} \nonumber \\&+ (4.3202) E_{AZI} + (1.6057) E_{ReZG_1} + (7.6624) E_{ReZG_2} + (36.3930) E_{ReZG_3}, \end{aligned}$$

12 $$\begin{aligned} \text {Multi. Linear Reg. : TPSA}&= 104.3100 + (37034.9815) E_{R_1} + (9668.6404) E_{R_{-1}} \nonumber \\&+ (16874.7756)E_{R_{1/2}}-(54374.9634) E_{R_{-1/2}} \nonumber \\&-(54375.3403) E_J + (17354.7896) E_{GA} + (24258.0159) E_{ABC} + (45163.9464) E_{M_1}\nonumber \\&+ (37034.9815) E_{M_2} + (-5794.9893) E_F + (-4960.5334) E_{HM} + (213.1249) E_{AZI} \nonumber \\&+ (11719.2140) E_{ReZG_1} + (-55383.7579) E_{ReZG_2} + (-24398.5301) E_{ReZG_3}, \end{aligned}$$

13 $$\begin{aligned} \text {Ridge Regression : HAC}&= 28.7000 + (1.1989) E_{R_1} + (-0.4720)E_{R_{-1}} \nonumber \\&+ (0.7580) E_{R_{1/2}} + (0.5440) E_{R_{-1/2}}+(0.5440) E_J \nonumber \\&+ (0.7124) E_{GA} + (0.6437) E_{ABC} + (0.8500) E_{M_1} \nonumber \\&+ (1.1989) E_{M_2}+ (1.5385) E_F + (1.3437) E_{HM} \nonumber \\&+ (0.5232) E_{AZI} + (0.3693) E_{ReZG_1}+ (0.6917) E_{ReZG_2} + (2.2080) E_{ReZG_3}, \end{aligned}$$

14 $$\begin{aligned} \text {Lasso Regression : HAC}&= 28.7000 + (11.8539) E_{ReZG_3}, \end{aligned}$$

15 $$\begin{aligned} \text {ElasticNet Regression : HAC}&= 28.7000 + (0.8651) E_{R_1} \nonumber \\&+ (0.5162) E_{R_{-1}} + (0.7719) E_{R_{1/2}} + (0.7276) E_{R_{-1/2}}+ (0.7277) E_J \nonumber \\&+ (0.7611) E_{GA} + (0.7466) E_{ABC} + (0.7915) E_{M_1} \nonumber \\&+ (0.8652) E_{M_2}+ (0.9375) E_F + (0.8967) E_{HM} \nonumber \\&+ (0.7131) E_{AZI} + (0.6888) E_{ReZG_1} + (0.7564) E_{ReZG_2} + (1.0758) E_{ReZG_3}, \end{aligned}$$

16 $$\begin{aligned} \text {Lasso Regression : C}&= 668.2000 + (-1529.9283) E_{R_{-1}}+ (619.6337) E_{R_{1/2}} + (515.4111) E_{ReZG_3}. \end{aligned}$$

## Discussion

Entropy measures are used to predict the physical and chemical properties of drugs or chemical compounds. In “Hyaluronic acid-paclitaxel conjugate”, we computed the reverse degree-based entropy measures for hyaluronic acid-paclitaxel conjugate for $s \ge 1$. Table 4 shows the numerical comparisons of reverse degree-based entropy measures for small values of *s* for hyaluronic acid-paclitaxel conjugate. Figure 4 demonstrates that all entropy measures exhibit an upward trend as the value of *s* increases. These results will be helpful to the pharmaceutical industry.

In “Statistical analysis of entropy measures”, we propose a statistical analysis of reverse degree-based entropy measures using the physical properties of cancer drugs. We find that the reverse degree-based entropy measures show a significant relationship with the physical properties. We employ Multiple Linear, Ridge, Lasso, Elastic Net, and Support Vector regression to examine the relationship between entropy measures and physical properties. All the computed results are listed in Tables 7, 8, 9, 10, 11, 12, 13, 14, 15, 16 and 17. Additionally, we propose the model for each relationship and include only the most significant models that will be used to estimate those physical properties that have not yet been calculated. We examine the following relationships:

- Table 7 presents a comparative analysis of various regression models, highlighting their differences in predictive capabilities. The results indicate that Multiple Linear Regression exhibits the weakest performance, characterized by a relatively low coefficient of determination ($R^2 = 0.228$) and the highest mean squared error (MSE = 100534.7691), suggesting a poorer fit and higher prediction errors. In contrast, Ridge and Lasso Regression demonstrate significant improvements, with $R^2$ values of 0.997 and 0.997, respectively. These models also exhibit substantially lower MSEs, indicating their superior ability to capture the relationship between entropy measures and the boiling point. Notably, ElasticNet Regression achieves a high $R^2$ of 0.998 and the lowest MSE among the models (3349.7946), striking a balance between prediction accuracy and generalization. Support Vector Regression (SVR) yields the highest MSE (8921.9773), indicating low predictive power, although its $R^2=0.986$ is slightly lower than the other models, implying a potential trade-off in explaining data variability. Overall, Lasso Regression is preferred for maximizing explanatory power while maintaining good prediction accuracy for boiling point. The Lasso Regression model for boiling point is presented in Eq. (4).
- Table 8 presents a comparative analysis of regression models, highlighting their differences in predictive capabilities. The results indicate that Multiple Linear Regression exhibits the weakest performance, characterized by a relatively low coefficient of determination and the highest mean squared error. This means the model is a poorer fit and has higher prediction errors. In contrast, Ridge, Lasso and ElasticNet Regression demonstrate significant improvements, with the same $R^2$ value of 0.998 and lower MSEs of 48.2737, 49.7108 and 51.3297, respectively. Ridge Regression achieves a high $R^2$ and the lowest MSE among the models, striking a balance between prediction accuracy and generalization. Support Vector Regression yields that $R^2=0.984$ is slightly lowest than the other models and the highest MSE (139.9596), indicating the model is a poorer fit and has higher prediction errors. Overall, Ridge Regression is preferred for maximizing explanatory power while maintaining good prediction accuracy for enthalpy of vaporization. Ridge Regression model for enthalpy of vaporization is presented in Eq. (5).
- Table 9 presents a comparative analysis of regression models for flash point. The results indicate that Multiple Linear Regression exhibits the weakest performance, characterized by a relatively low coefficient of determination and the highest mean squared error. In contrast, Lasso Regression demonstrates significant improvements, with $R^2$ value of 0.938 and lower MSE of 25416.2368. On the other hand, Ridge, ElasticNet, and Support Vector Regression yield that $R^2$ values are slightly lower than the Lasso Regression and the highest MSEs, indicating the models are poorer fit and have higher prediction errors. Overall, Lasso Regression is preferred for maximizing explanatory power while maintaining good prediction accuracy for flash point. The Lasso Regression model for flash point is presented in Eq. (6).
- Table 10 presents a comparative analysis of regression models for molar refractivity. The results indicate that Multiple Linear Regression exhibits the weakest performance, characterized by a relatively low coefficient of determination and the highest mean squared error. In contrast, Ridge and Lasso Regression demonstrate significant improvements, with $R^2$ values of 0.813 and 0.824, respectively and with lower MSEs. Notably, ElasticNet Regression achieves a high $R^2$ of 0.825 and the lowest MSE among the models (250.6917), striking a balance between prediction accuracy and generalization. Support Vector Regression (SVR) yields the highest MSE (1256.6219), indicating low predictions, although its $R^2$ is slightly lower than the other models, implying a potential trade-off in explaining data variability. Overall, ElasticNet is preferred for maximizing explanatory power while maintaining good prediction accuracy for molar refractivity. ElasticNet Regression model for molar refractivity is presented in Eq. (7).
- Table 11 presents a comparative analysis of regression models for molar volume. The results indicate that Multiple Linear, Ridge, ElasticNet and vector Support Regression exhibit the weakest performances, characterized by a relatively low coefficient of determination and the highest mean squared errors. In contrast, Lasso Regression demonstrates significant improvements, with a $R^2$ value of 1.000 and with lower MSE among the models, striking a balance between prediction accuracy and generalization. Overall, Lasso Regression is preferred for maximizing explanatory power while maintaining good prediction accuracy for molar volume. The Lasso Regression model for molar volume is presented in Eq. (8).
- Table 12 presents a comparative analysis of regression models for polarization. The results indicate that Multiple Linear and Support Vector Regression exhibits the weakest performances, characterized by a relatively low coefficient of determination and the highest mean squared error. In contrast, Ridge, Lasso and ElasticNet Regression demonstrate significant improvements, with $R^2$ values of 0.817, 0.828 and 0.829, and lower MSEs of 39.9896, 39.0514 and 39.6940, respectively. Lasso Regression achieves the lowest MSE among the models, striking a balance between prediction accuracy and generalization. Overall, Lasso Regression is preferred for maximizing explanatory power while maintaining good prediction accuracy for polarization and no multicollinearity. Ridge Regression model for polarization is presented in Eq. (9).
- Table 13 presents a comparative analysis of regression models for molecular weight. The results indicate that Multiple Linear, Lasso, and Vector Support Regression exhibits the weakest performances, characterized by the highest mean squared errors. In contrast, Ridge Regression and ElasticNet demonstrate significant improvements, with $R^2$ values of 1.000 and 0.999 with lower MSEs 885.8908 and 897.8231 among the models, striking a balance between prediction accuracy and generalization. Overall, Ridge Regression is preferred for maximizing explanatory power while maintaining good prediction accuracy for molecular weight. Ridge Regression model for molecular weight is presented in Eq. (10).
- Table 14 presents a comparative analysis of regression models for monoisotopic mass. The results indicate that Multiple Linear, Lasso, and Vector Support Regression exhibits the weakest performances, characterized by the highest mean squared errors. In contrast, Ridge and ElasticNet Regression demonstrate significant improvements, with $R^2$ values of 1.000 and 0.999 with lower MSEs 887.2696 and 899.1999 among the models, striking a balance between prediction accuracy and generalization. Overall, Ridge Regression is preferred for maximizing explanatory power while maintaining good prediction accuracy for monoisotopic mass. Ridge Regression model for monoisotopic mass is presented in Eq. (11).
- Table 15 presents a comparative analysis of regression models for topological polar surface area. The results indicate that Multiple Linear Regression exhibits the highest performance, characterized by a relatively high coefficient of determination ($R^2=996$). In contrast, Ridge, Lasso, ElasticNet and Support Vector Regression demonstrate the lowest performance, characterized by a relatively low coefficient of determination. Overall, Multiple Linear Regression is preferred for maximizing explanatory power while maintaining good prediction accuracy for topological polar surface area. Multiple Linear Regression model for topological polar surface area is presented in Eq. (12).
- Table 16 presents a comparative analysis of regression models for heavy atom count. The results indicate that Multiple Linear and Support Vector Regression exhibits the weakest performances, characterized by a relatively low coefficient of determination and the highest mean squared error. In contrast, Ridge, Lasso and ElasticNet Regression demonstrate significant improvements, with $R^2$ values of 0.995, 0.996 and 0.996, and lower MSEs of 8.9858, 9.0149 and 9.2303, respectively. Lasso Regression achieves the highest $R^2$ value among the models, striking a balance between prediction accuracy and generalization. Overall, Lasso Regression is preferred for maximizing explanatory power while maintaining good prediction accuracy for heavy atom count. Models for heavy atom count are presented in Eqs. (13)–(15). However, the best model is the Lasso Regression model (Eq. 15), as it does not exhibit multicollinearity.
- Table 17 presents a comparative analysis of regression models for complexity. The results indicate that Multiple Linear, Ridge, ElasticNet and Vector Support Regression exhibits the weakest performances, characterized by low coefficient of determination and highest mean squared errors. In contrast, Lasso Regression demonstrates significant improvements, with $R^2$ value of 0.993 with lower MSE 11751.3839 among the models, striking a balance between prediction accuracy and generalization. Overall, Lasso Regression is preferred for maximizing explanatory power while maintaining good prediction accuracy for complexity. The Lasso Regression model for complexity is presented in Eq. (16).

Based on the above results and their explanations, we can conclude that the physical properties, such as boiling point, enthalpy of vaporization, flash point, molar refractivity, molar volume, polarization, molecular weight, monoisotopic mass, topological polar surface area, and complexity, can be predicted using the reverse entropy measures.

## Conclusion

Entropy measures are utilized to predict physical and chemical properties of drugs. In this study, we computed entropy measures for the hyaluronic acid-paclitaxel conjugate. The results exhibited numerical values that demonstrated the effectiveness of entropy measures. The utilization of reverse degree-based entropy measures proved valuable in quantitative structure-property relationship (QSPR) investigations as predictive measures. This investigation focused on assessing the predictive ability of entropy measures by analyzing the physical properties of cancer drugs. The obtained findings demonstrated a robust positive correlation between the boiling point, enthalpy of vaporization, flash point, molar refractivity, molar volume, polarization, molecular weight, monoisotopic mass, topological polar surface area, complexity, and entropy measures. Our analysis determined that the entropy measures $\mathscr {E}_{R_{1}}$, $\mathscr {E}_{R_{-1}}$, $\mathscr {E}_{R_{\frac{1}{2}}}$, $\mathscr {E}_{R_{\frac{-1}{2}}}$, $\mathscr {E}_{ABC}$, $\mathscr {E}_{GA}$, $\mathscr {E}_{M_1}$, $\mathscr {E}_{M_2}$, $\mathscr {E}_{AZI}$, $\mathscr {E}_{HM_1}$, $\mathscr {E}_{F}$, $\mathscr {E}_{J}$, $\mathscr {E}_{ReZG_1}$, $\mathscr {E}_{ReZG_2}$, and $\mathscr {E}_{ReZG_3}$ can be used for predicting physical properties. We developed a predictive model for each relationship and selected only the most significant models to estimate physical properties that have not yet been calculated.

## Data Availability

All data generated or analysed during this study are included in this published article.
